# Supplementary figures and images for: A biomechanical analysis of the stand-up paddle board stroke: a comparative study
Source: PeerJ. 2019 Nov 1;7:e8006. doi: 10.7717/peerj.8006 (PMC6827442; doi:10.7717/peerj.8006)

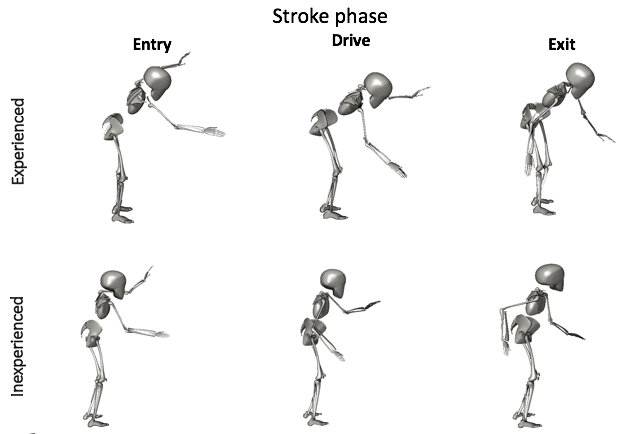

Supplement: Supplemental Information 1 [file peerj-07-8006-s001.docx]

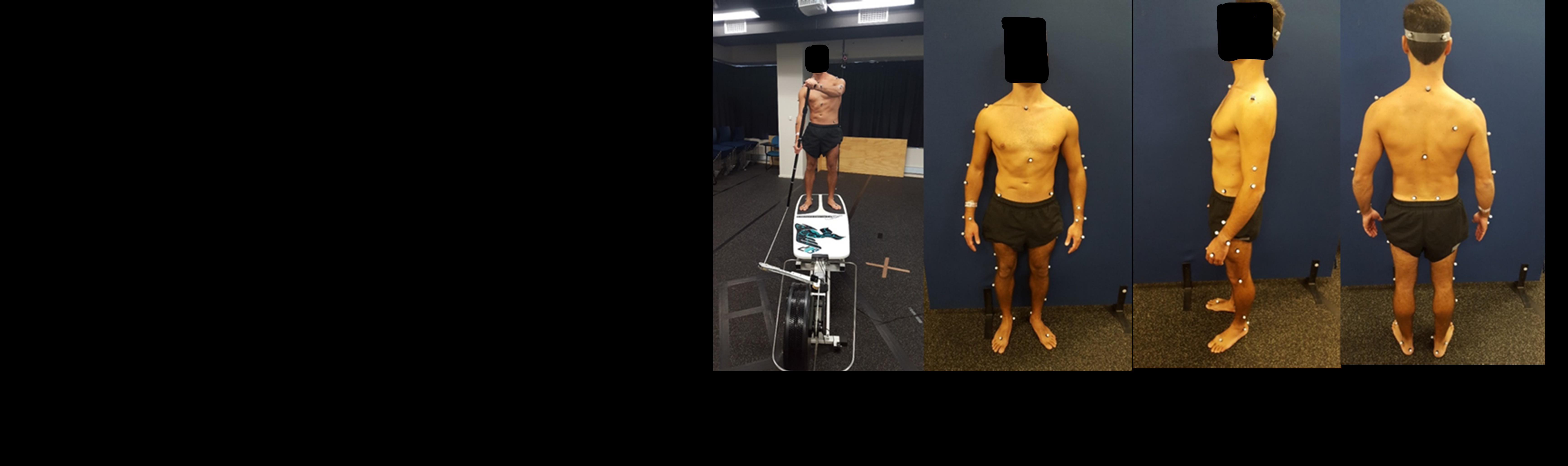

Supplement: Supplemental Information 2 [file peerj-07-8006-s002.jpg]
